# *In vitro* fermentation properties of magnesium hydride and related modulation effects on broiler cecal microbiome and metabolome

**DOI:** 10.3389/fmicb.2023.1175858

**Published:** 2023-08-09

**Authors:** Heng Hu, He Zhu, Haiyan Yang, Wen Yao, Weijiang Zheng

**Affiliations:** ^1^College of Animal Science and Technology, Nanjing Agricultural University, Nanjing, Jiangsu, China; ^2^Center of Hydrogen Science, Shanghai Jiao Tong University, Shanghai, China; ^3^Key Lab of Animal Physiology and Biochemistry, Ministry of Agriculture, Nanjing, Jiangsu, China

**Keywords:** magnesium hydride, microbiota, metabolites, broiler caecum, gas production, *in vitro* fermtation

## Abstract

Magnesium hydride (MGH), a highly promising hydrogen-producing substance/additive for hydrogen production through its hydrolysis reaction, has the potential to enhance broiler production. However, before incorporating MGH as a hydrogen-producing additive in broiler feed, it is crucial to fully understand its impact on microbiota and metabolites. *In vitro* fermentation models provide a fast, reproducible, and direct assessment tool for microbiota metabolism and composition. This study aims to investigate the effects of MGH and coated-magnesium hydride (CMG) on fermentation characteristics, as well as the microbiota and metabolome in the culture of *in vitro* fermentation using cecal inocula from broilers. After 48 h of incubation, it was observed that the presence of MGH had a significant impact on various factors. Specifically, the content of N-NH_3_ decreased, while the total hydrogen gas and total SCFAs increased. Furthermore, the presence of MGH promoted the abundance of SCFA-producing bacteria such as *Ruminococcus*, *Blautia*, *Coprobacillus*, and *Dysgonomonas*. On the other hand, the presence of CMG led to an increase in the concentration of lactic acid, acetic acid, and valeric acid. Additionally, CMG affected the diversity of microbiota in the culture, resulting in an enrichment of the relative abundance of Firmicutes, as well as genera of *Lactobacillus*, *Coprococcus*, and *Eubacterium*. Conversely, the relative abundance of the phylum Proteobacteria and pathogenic bacteria *Shigella* decreased. Metabolome analysis revealed that MGH and CMG treatment caused significant changes in 21 co-regulated metabolites, primarily associated with lipid, amino acid, benzenoids, and organooxygen compounds. Importantly, joint correlation analysis revealed that MGH or CMG treatments had a direct impact on the microbiota, which in turn indirectly influenced metabolites in the culture. In summary, the results of this study suggested that both MGH and coated-MGH have similar yet distinct positive effects on the microbiota and metabolites of the broiler cecal in an *in vitro* fermentation model.

## Introduction

The rapid genetic selection in poultry breeding has significantly improved efficiency in terms of feed conversion and growth rate (Zaboli et al., 2019). However, the intensified selection has rendered poultry birds more susceptible to environmental and nutritional stresses. At the molecular level, it has been observed that these stresses often lead to the overproduction of free radicals and oxidative stress (Sies et al., 2017). Consequently, birds exposed to unfavorable environmental or inadequate nutritional conditions are prone to oxidative stress, resulting in biological dysfunction, organ damage, compromised growth performance, and reduced production (Gonzalez-Rivas et al., 2020). To address the modulation of antioxidant defenses in poultry, various feed ingredients and supplements containing antioxidant have been formulated to support chicken health, productivity, and meat quality (Surai and Kochish, 2019). However, the conventional use of synthetic antioxidants raises concerns among consumers due to potential overuse and the presence of residues that may pose risk to human health (Botterweck et al., 2000). Therefore, there is an immediate need for research focus on the development of novel and safe antioxidant agents.

Interestingly, molecular hydrogen has emerged as a novel biological agent with preventive and therapeutic effects on various organs, attributed to its antioxidative, anti-inflammatory and antiapoptotic properties (Tian et al., 2021). Unlike other organic or synthetic antioxidants, molecular hydrogen does not generate oxidized molecules following reduction. This unique characteristic makes molecular hydrogen as an ideal candidate for antioxidant applications in the poultry industry (Ichihara et al., 2015). Several approaches have been employed to administer molecular hydrogen to animals, including hydrogen gas inhalation, injection or administration of hydrogen-rich water/saline, and the used of hydrogen-producing bacteria or prebiotic (Chen et al., 2013; Zheng et al., 2018). Hydrogen-rich water, which typically contains a high concentration of dissolved hydrogen (0.4–0.9 ppm), can be conveniently obtained through electrolyzed reduced water (ERW). Notably, a study demonstrated that electrolyzed reduced water partially alleviated skeletal muscle oxidative stress induced by reactive oxygen species (ROS) and improved growth performance in broiler chickens subjected to medium-term chronic heat treatment (5 days at 34°C) (Azad et al., 2013). Additionally, in layers exposed to long-term chronic heat stress (42 days at 34°C), drinking ERW improved villus length, villus length/crypt depth ratio in the jejunum, enhanced plasma antioxidative enzyme activities, reduced plasma malondialdehyde levels, and significantly improved production performance (egg production and feed conversation ratio) and egg quality (egg thickness) (Zhang et al., 2022). Moreover, supplementation with hydrogenated water (HNW) increased antioxidant enzyme activity in broiler chickens (Shin et al., 2016). However, the high diffusivity and low solubility of molecular hydrogen necessitate specialized equipment and accessories for hydrogen gas inhalation, as well as specific measures for the administration of hydrogen-rich water in poultry industry. Thus, identifying novel hydrogen-producing additives may offer a more feasible and efficient approach to delivering molecular hydrogen to broiler birds.

It is worth noting that magnesium hydride (MgH_2_) has shown promise as a hydrogen-producing substance/additive through its hydrolysis reaction (Hirscher et al., 2020). Studies have demonstrated that orally administration of MgH_2_ (at a dose of 0.9 mg/kg) increased blood hydrogen levels and decreases plasma triglyceride concentration in rats, leading to an extension of their average lifespan (Kamimura et al., 2016). Moreover, the introduction of MgH_2_ to aging or short-lived flies has been found to enhance their survival, delay the onset of intestinal barrier dysfunction, and significantly improve physical activity levels (Klichko et al., 2019). However, the hydrolysis reaction kinetics of MgH_2_ in pure water is exceptionally slow, necessitating measurements to enhance the reaction and facilitate its application in feed (Chao, 2018). Importantly, precise control of the release of molecular hydrogen from MgH_2_ is essential to efficiently deliver hydrogen to different regions of the intestine, allowing the design of specialized hydrogen-producing additive for various purposes in feed. In this regard, encapsulation techniques such as microencapsulation can assist to solve the potential concerns mentioned above.

The role of the intestinal microbiota and its metabolites shaping the health and productive of chickens is widely recognized (Aruwa et al., 2021; Bindari and Gerber, 2022). Several factors, including diet, feed additives, and the microenvironment, has been shown to influence the composition and function of the gut microbiome (Diaz Carrasco et al., 2019). Thus, before incorporating the hydrogen-producing additive MgH_2_ into the broiler feed, it is crucial to fully understand the effects of MgH_2_ and its microencapsulation on the microbiota and metabolites in the intestine of broilers. Molecular hydrogen (H_2_) is a significant byproduct produced by many gut bacteria, and microbes capable of utilizing it as a substrate may have evolved an advantage in the anaerobic gut ecosystem (Hylemon et al., 2018). However, the impacts of exogenous H_2_ delivered from MgH_2_ on the gut microbiota remains unexplored. Therefore, in this study, we hypothesized that MGH can modulate the composition and functions of intestinal microorganisms through the producing hydrogen gas. As a result, our study aims to examine the effects of MgH_2_ and its microencapsulation on the cecal microbiota and metabolome of broiler chicken, utilizing an *in vitro* fermentation model.

## Materials and methods

### Experimental design

This study was conducted in two separate fermentation runs, with a five-week gap between them. The study design included four experimental groups, with each group consisting of five replicates. These groups were as follows: blank group (medium without any substrate or additive), CON group (medium supplemented with 0.5 g of feed), MGH group (medium supplemented with 0.5 g feed and 200 mg/L of MgH_2_), and CMG group (medium supplemented with 0.5 g of feed and 400 mg/L of coated MgH_2_), respectively.

### *In vitro* fermentation model

#### Culture medium

Our *in vitro* anaerobic mixed culture experiment was based on the method previously described (Donalson et al., 2008). The culture used was an anaerobic dilution solution (ADS) consisting of 0.45 g/L K_2_HPO_4_, 0.45 g/L KH_2_PO_4_, 0.45 g/L (NH_4_)_2_SO_4_, 0.9 g/L NaCl, 0.1875 g/L MgSO_4_-7H_2_O, 0.12 g/L CaCl_2_-2H_2_O, 1 mL/L 0.1% resazurin, 0.05% cysteine-HCl, and 0.4% CO_2_-saturated sodium carbonate, with the sodium carbonate added last as previously described (Donalson et al., 2008). ADS was sparged with a CO_2_ gas for 120 min using an airstone prior to bottling and autoclaving. Autoclaved ADS was cooled to room temperature and allowed to equilibrate overnight.

#### Cecal microbiota inoculum

The cecal microbiota inoculum used in the *in vitro* fermentation was obtained from five male Archer Abor broilers at 37 days old. These broiler chickens were fed a commercial feed and has not received any antibiotics treatment prior to collection. Additionally, they were not feed-restricted. During the collection process, the ceca were removed, and the contents without tissue were individually collected in sterile and anaerobic containers. Equal amounts of cecal contents from each bird were then weighed and combined by wet weight. The combined contents were subsequently diluted 1:300 with pre-warmed (37°C) sterile ADS. To ensure proper mixing, the diluted mixture was homogenized for the 60s and then filtered through a fourfold sterile cheesecloth. The resultant filtrate served as the inoculum for the fermentation experiment. Throughout those procedures, a constant stream of CO_2_ was maintained to ensure anaerobic conditions.

#### *In vitro* fermentation

The *in vitro* fermentation experiment followed a modified version of the protocol previously described (Donalson et al., 2008). Each fermentation bottle contained 0.5 g of broiler commercial feed as substrate and 45 mL of ADS culture medium. Two different additives were used in separate bottles: MGH powder (Shanghai Magnesium Source Power Technology Co., LTD) and coated MGH (50% coated magnesium hydride synthesized by King Techina Feed Co., LTD). Before adding the inoculum, the appropriate additive was added to its corresponding bottle. The final diluted cecal inoculum (1,300) was then added to the solution in a 1:10 ratio (v/v), resulting in a final volume of 50 mL. For the control group, the bottles contained only the ADS culture and the inoculum. To ensure anaerobic conditions, the fermentation bottles were closed with rubber stoppers and aluminum caps. Subsequently, they were incubated at a temperature of 37°C for a total duration of 48 h. During the fermentation process, measurements of air pressure and gas production were taken at specific time intervals. These time points included 3, 6, 9, 12, 14, 17, 20, 24, 30, 35, and 48 h of fermentation. The pressure transducer technique was employed to measure air pressure and gas production within the fermentation bottles (Theodorou et al., 1995).

#### Sample collection

At the end of the 48-h fermentation period, the cultures in the bottles were stopped, and various measurements and sample collections were performed. Firstly, the pH of each fermentation culture was measured. Additionally, two sample tubes were collected from each bottle. These sample tubes, containing the fermentation culture, were immediately frozen in liquid nitrogen, and then stored at −80°C for the subsequent analysis. The analysis included the measurement of short-chain fatty acids (SCFAs), 16sRNA sequencing, and metabolomics. Furthermore, the substrate present in each fermentation bottle was collected for analysis of dry matter (DM) disappearance.

#### Analyses

All substrates were analyzed for their DM contents (GB/T6435-2014). The pH (pH meter, Hanna Instruments, Limena, Italy) of the post-fermentative samples was also determined. SCFAs concentrations in the fermentation liquids were analyzed by gas chromatography (GC-14A with an FID detector; Shimadzu, Japan; capillary column: 30 m × 0.32 mm × 0.25 μm film thickness) with a H_2_ flame ionization detector and split injection as previously described (Mao et al., 2007). The column, injector, and detector temperatures were 140, 180, and 180°C, respectively. The concentration of ammonia-N in the fermented liquid was analyzed by the colorimetric according to the method as previously described (Shen et al., 2017). The lactic acid content was determined by a commercial kit (Nanjing Jiancheng Bioengineering Institute, Nanjing, China) and the operation was strictly according to the commercial kit instructions.

#### Gas production kinetics

The organic matter cumulative gas production, measured in mL of gas produced per gram of OM weighed into the fermentation bottle, was fitted to the monophasic model described by Groot et al. (1996). The equation for the model is as follows: $Y=\overset{n}{\underset{i=1}{\sum }}\frac{A_{i}}{1+\left(C_{i}/t\right)Bi}\;$ , in this equation, Y = the cumulative gas production (mL/g DM); A_i_ = asymptotic gas production for phase i (mL); B_i_ = smoothness factor for phase i; C_i_ = time at which half of the asymptote gas has been produced for phase i(h); i = number of phase in gas production; t = time (h). Additionally, the maximum rate of gas production (R_max_) and the time at which it occurs (T_Rmax_) were calculated as previously reported (Bauer et al., 2001): R_max_ = {A×(C^B^) × B×(T_Rmax_^(−B − 1)^)}/{1 + (C^B^) × (T_Rmax_^(−B)^)}^2^ and T_Rmax_ = C × {(B − 1)/(B + 1)}^(1/B)^, respectively.

#### Determination of hydrogen gas

During the measurement of gas production, the gases from each serum bottle were collected using a syringe and transferred into a multi-layer foil sampling bag (Dalian Delin Gas Packing Co., Ltd., Dalian, China) for later analysis. To measure the hydrogen production in the collected has samples at different timepoints, a combination of a gas-tight plastic chamber (0.325 m*0.225 m*0.155 m) and a hydrogen gas detector (Ennix GS40 Ennix Gmbh, Eichingen, Germany) was utilized. The hydrogen volume was calculated using the following formula: Hydrogen detector reading (ppm)* Volume of the gas-tight plastic chamber (m3) = Volume of hydrogen in the gas-tight plastic chamber (mL).

### Microbial community analysis

#### Genomic DNA extraction

The extraction of total genomic DNA from the fermentation cultures was performed using DNeasy Power Soil Kit (QIAGEN, New York, USA) according to the manufacturer’s protocols. The quality and quantity of extracted DNA were detected using a NanoDrop ND-1000 spectrophotometer (Thermo Fisher Scientific, Waltham, MA, USA) and agarose gel electrophoresis, respectively.

#### Microbiota profiling with 16S rRNA amplicon sequencing

The microbiota community in the culture samples was analyzed using the primers 338F (5’-ACTCCTACGGGAGGCAGCA-3′) and 806R (5’-GGACTACHVGGGTWTCTAAT-3′) (Parada et al., 2016). These primers specifically target the V3–V4 region of the ribosomal RNA gene. After the PCR amplification step, the resulting amplicons were purified with Agencourt AMPure Beads (Beckman Coulter, Indianapolis, IN). The quantification of the purified PCR amplicons was performed using PicoGreen dsDNA Assay Kit (Invitrogen, Carlsbad, CA, USA). Following individual quantification, the purified amplicons were pooled in equal amounts, and pair-end 2 × 300 bp sequencing was performed using the Illumina Miseq 250 platform (Illumina Inc. San Diego, CA, United States) at Suzhou PANOMIX Biomedical Tech Co., LTD.

#### Microbial metabolite analysis

Metabolome analyses in this study were conducted by PANOMIX (Suzhou, China). Briefly, 400 μL of methanol was added into 1.5 mL of the metabolic sample. Then, the sample was mixed for 1 min, followed by centrifugation at 12,000 rpm for 10 min. The supernatant was carefully transferred into a new 2 mL centrifuge tube, concentrate, and dried. Subsequently, 150 μL of 2-chloro-l-phenylalanine (4 ppm) solution, prepared using 80% methanol water and stored at 4°C, was added to re-dissolve the sample. The supernatant was filtered by a 0.22 μm membrane and transfer to a detection bottle for analysis using Liquid Chromatograph Mass Spectrometer (LC–MS) detection. The LC analysis was conducted using a Vanquish UHPLC System (Thermo Fisher Scientific, USA). Chromatography was performed with an ACQUITY UPLC ^®^ HSS T3 (150 × 2.1 mm, 1.8 μm) (Waters, Milford, MA, USA). The column temperature was maintained at 40°C. The flow rate was set at 0.25 mL/min, and the injection volume was 2 μL. The mass spectrometric detection of metabolites was carried out using a Q Exactive instrument (Thermo Fisher Scientific, New York, USA) with ESI ion source. Simultaneous MS1 and MS/MS (Full MS-ddMS2 mode, data-dependent MS/MS) acquisition modes were utilized. For more detailed information regarding the extraction and LC–MS analysis procedures, please refer to the study previously conducted (Sun et al., 2022).

### Statistical analysis

The data were presented as means ± SEM and were analyzed using *t*-test or one-way ANOVA (analysis of variance) with Duncan’s test for determining significant differences among groups. All statistical analyses were conducted using SPSS (IBM SPSS 25.0, Chicago, IL, USA). For multivariate data analyses and modeling, the Ropls software (Boulesteix and Strimmer, 2007) was utilized. Prior to analysis, the data were mean-centered using scaling. Principal component analysis (PCA), orthogonal partial least-square discriminant analysis (PLS-DA), and partial least-square discriminant analysis (OPLS-DA) were constructed. Metabolic pathway analysis was performed using MetaboAnalyst 5.0^1^ and KEGG^2^ based on the identified cecal metabolites with significant differences (*p* < 0.05). To analyze the relationship involving the total hydrogen production, changes in the microbiota, and differentially expressed metabolites, Spearman’s rank correlation coefficient was employed. The correlation analysis was performed using Cytoscape 3.9.1 software. OmicStudio online tools^3^ and GraphPad Prism 8.0 (GraphPad Software Inc., San Diego, CA, USA) were employed for generating the figures.

## Results

### Cumulative gas production kinetics and hydrogen gas production

**Figure 1:**
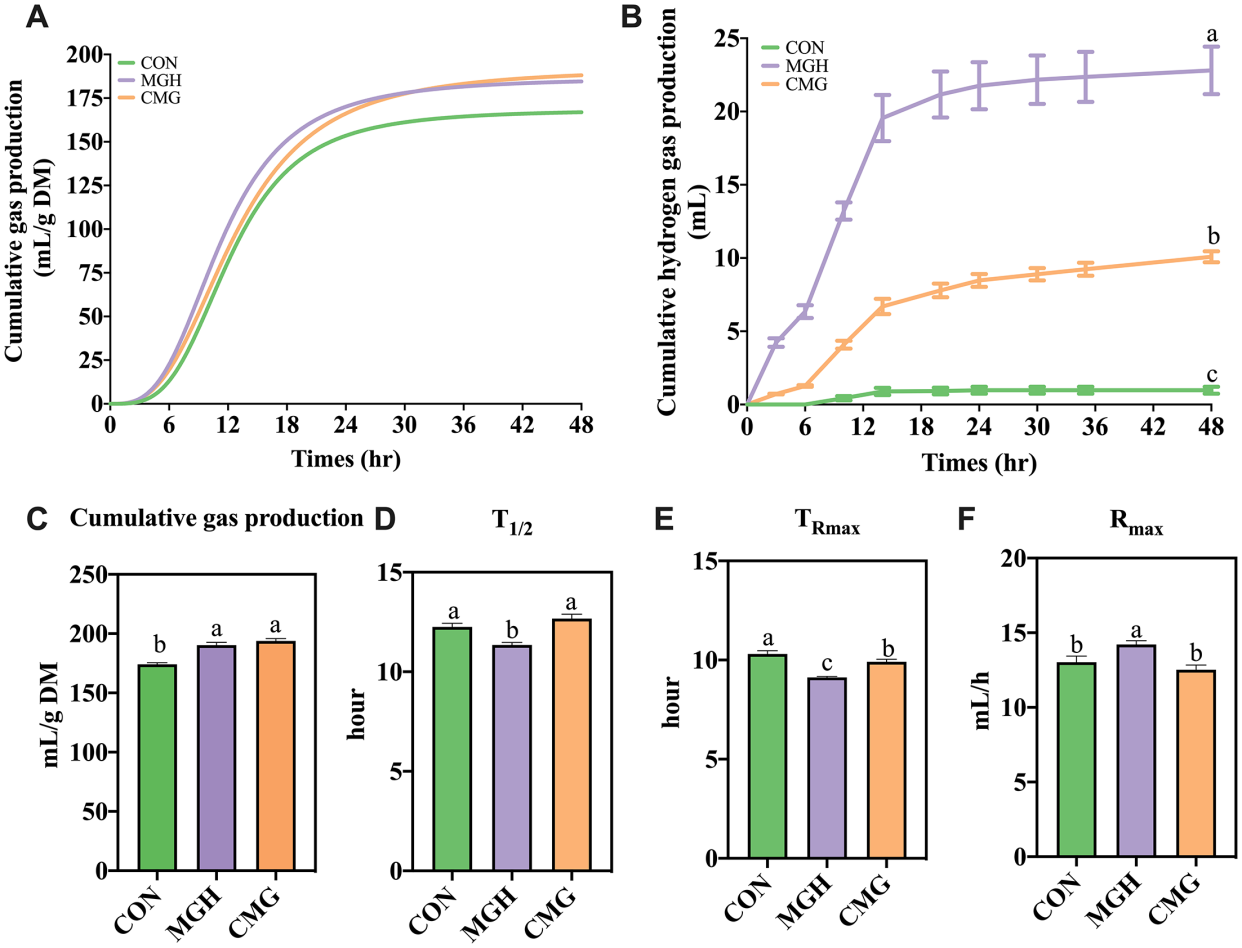
Effects of magnesium hydride (MGH) and coated-magnesium hydride (CMG) on the in vitro fermentation properties using cecal inocula from broilers. **(A)** The curves of cumulative gas production; **(B)** Curves of the cumulative hydrogen gas production; **(C)** Cumulative gas production; **(D)** T1/2; **(E)** TR_max_; and **(F)** R_max_. Data are means ±SEM (5 replications). Different lowercase letters indicate significant differences (*p*<0.05). CON, the control group, cecal content and feed; MGH, the magnesium hydride group, cecal content, feed and 200 mg/L MgH_2_; CMG, the coated- magnesium hydride group, cecal content, feed and 400 mg/L coated MgH_2_.

Figure 1A displays representative curves of the cumulative total gas profiles observed during a 48-h incubation period. The cumulative gas production in both the MGH and CMG groups were significantly higher compared to the CON group (*p* < 0.05; Figure 1C). However, there was no significant difference in these parameters between the MGH and CMG groups (*p* > 0.05). Regarding other fermentation kinetics parameter, the MGH treatment exhibited the highest R_max_ and shortest T_1/2_ (*p* < 0.05) compared to the CON and CMG groups (*p* < 0.05; Figures 1D,F). However, there was no significant difference in those parameters between the CON and CMG groups (*p* > 0.05). In terms of *T*_Rmax_ (Figure 1E), the MGH group had the earliest *T*_Rmax_ compared to both the CMG and CON groups (*p* < 0.05). Additionally, the CMG group also had an earlier *T*_Rmax_ compared to the CON group (*p* < 0.05).

To investigate the hydrogen-leasing ability of MGH and CMG groups in the cultures, the levels of H_2_ in sampled gas were measured at different timepoint. Figure 1B displays the cumulative hydrogen gas production curves for the MGH and CMG groups during *in vitro* fermentation, indicating that both groups produced a higher amount of hydrogen gas compared to the CON group. Regarding the total hydrogen gas production during the 48-h incubation period, the MGH group exhibited the highest volume of hydrogen gas (15.36 ± 0.14 mL) compared to the other groups (*p* < 0.05). The CMG group (5.72 ± 1.22 mL) also produced more hydrogen gas than the CON group (0.61 ± 0.14 mL) (*p* < 0.05).

### pH, DM, N-NH_3_, and lactic acid

As shown in Table 1, after 48 h of *in vitro* fermentation, the pH value in the cultures of MGH and CMG groups was significantly higher compared to the CON group (*p* < 0.05), while no difference was found between the MGH and CMG groups (*p* > 0.05). The DM disappearance rate in the CMG group cultures was significantly lower compared to the CON and MGH groups (*p* < 0.05). Nevertheless, there was no difference between the CON and MGH groups (*p* > 0.05). In terms of lactic acid levels, the CMG treatment led to a significant increase compared to the MGH and CON groups (*p* < 0.05). However, no significant difference was found between the CON and MGH groups (*p* > 0.05). The MGH group exhibited a significant decrease in N-NH_3_ levels compared to the CMG and CON groups (*p* < 0.05), while no difference was found between the CON and CMG groups (*p* > 0.05).

**Table 1:** Effects of magnesium hydride (MGH) and coated-magnesium hydride (CMG) on the pH, levels of N-NH_3_ and lactic acid, dry matter degradability in the culture of *in vitro* fermentation using cecal inocula from broilers (*n* = 5).

Item	CON^1^	MGH^2^	CMG^3^	SEM^4^	*p*-Value
pH	5.73^c^	5.99^a^	5.88^b^	0.11	<0.001
*In vitro* dry matter digestibility (%)	86.62^a^	87.35^a^	83.75^b^	0.40	<0.001
Lactic acid (mmol/L)	1.12^b^	1.18^b^	1.43^a^	0.05	0.004
N-NH_3_ (mmol/L)	11.19^a^	10.16^b^	11.05^a^	0.17	0.016

Means within a row, with no common superscript (a, b, c), differed significantly (*P* < 0.05). ^1^CON, the control group, cecal content and feed. ^2^MGH, the magnesium hydride group, cecal content, feed and 200 mg/l of MgH_2_. ^3^CMG, the coated-magnesium hydride group, cecal content, feed and 400 mg/l of coated MgH_2_. ^4^SEM, standard error of the mean.

### SCFAs production

**Figure 2:**
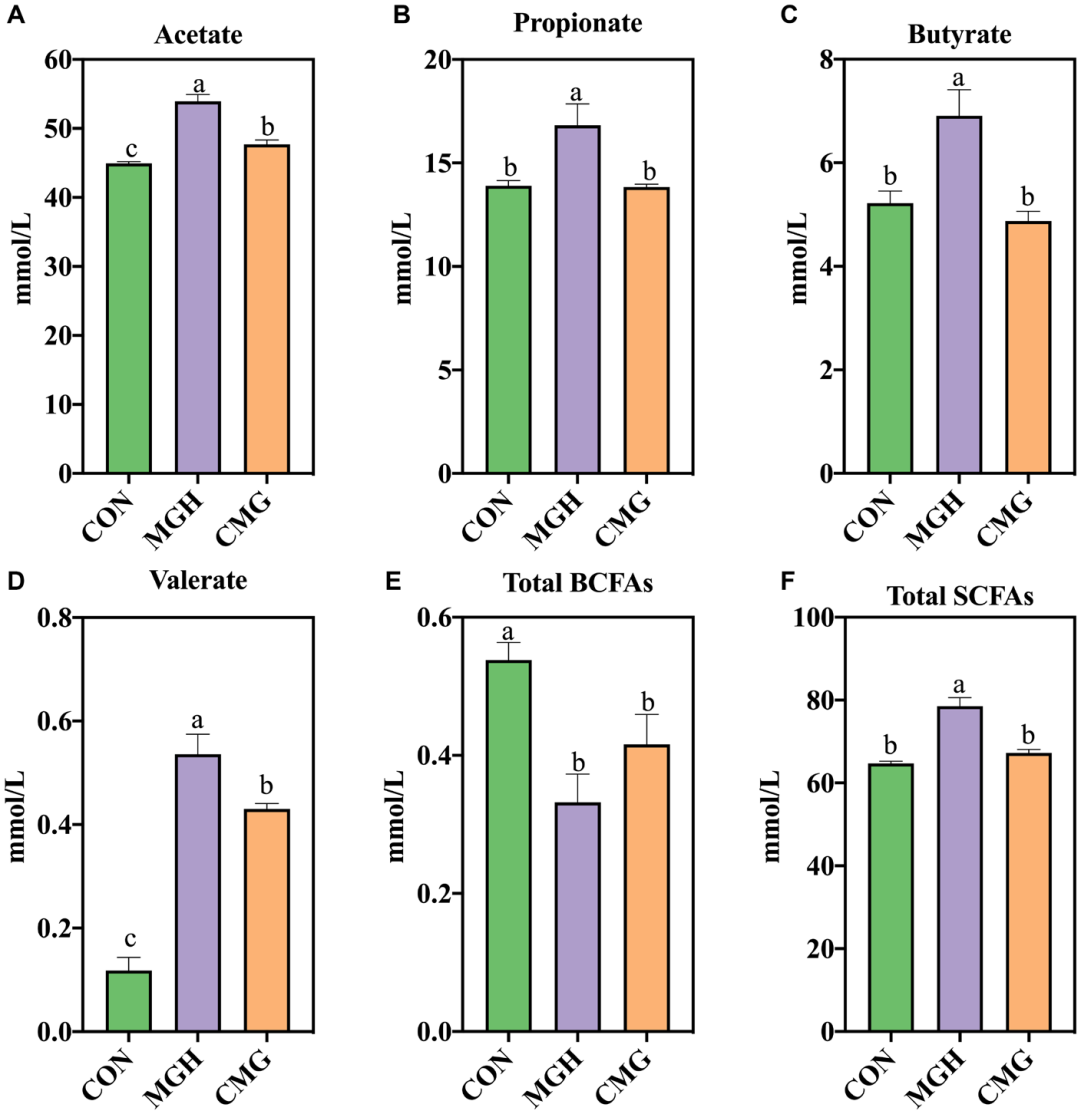
Effects of magnesium hydride (MGH) and coated-magnesium hydride (CMG) on short-chain fatty acids (SCFAs) in the culture of *in vitro* fermentation using cecal inocula from broilers. **(A)** Acetate; **(B)** Propionate; **(C)** Butyrate; **(D)** Valerate; **(E)** Total BCFAs; and **(F)** Total SCFAs. BCFA, branched chain fatty acids (sum of isobutyric and isovaleric acids). Data are means ±SEM (5 replications). Different lowercase letters indicate significant differences (*p*<0.05). CON, the control group, cecal content and feed; MGH, the magnesium hydride group, cecal content, feed and 200 mg/L MgH_2_; CMG, the coated- magnesium hydride group, cecal content, feed and 400 mg/L coated MgH_2_.

Figure 2 demonstrates that the supplementation of either MGH or CMG had a significant impact on the levels of SCFAs in the culture of *in vitro* fermentation using broiler cecal digesta. The MGH group exhibited significantly increased concentrations of propionate, butyrate, and total SCFAs in the culture compared to the CON and CMG groups (*p* < 0.05), while no difference was found between the CON and CMG groups (*p* > 0.05) (Figures 2B,C,F). Furthermore, compared to the CON group, both the MGH and CMG groups showed significantly increased levels of acetate and valerate (*p* < 0.05) (Figures 2A,D). Additionally, the MGH group had higher levels of acetate and valerate compared to the CMG group (*p* < 0.05). On the contrary, the branched-chain fatty acids (BCFAs) in the MGH and CMG was significantly lower than that in the CON group (*p* < 0.05) (Figure 2E), while no difference was found between the MGH and CMG groups (*p* > 0.05).

### Changes in microbial communities in culture

#### α diversity of microbiota in culture

**Figure 3:**
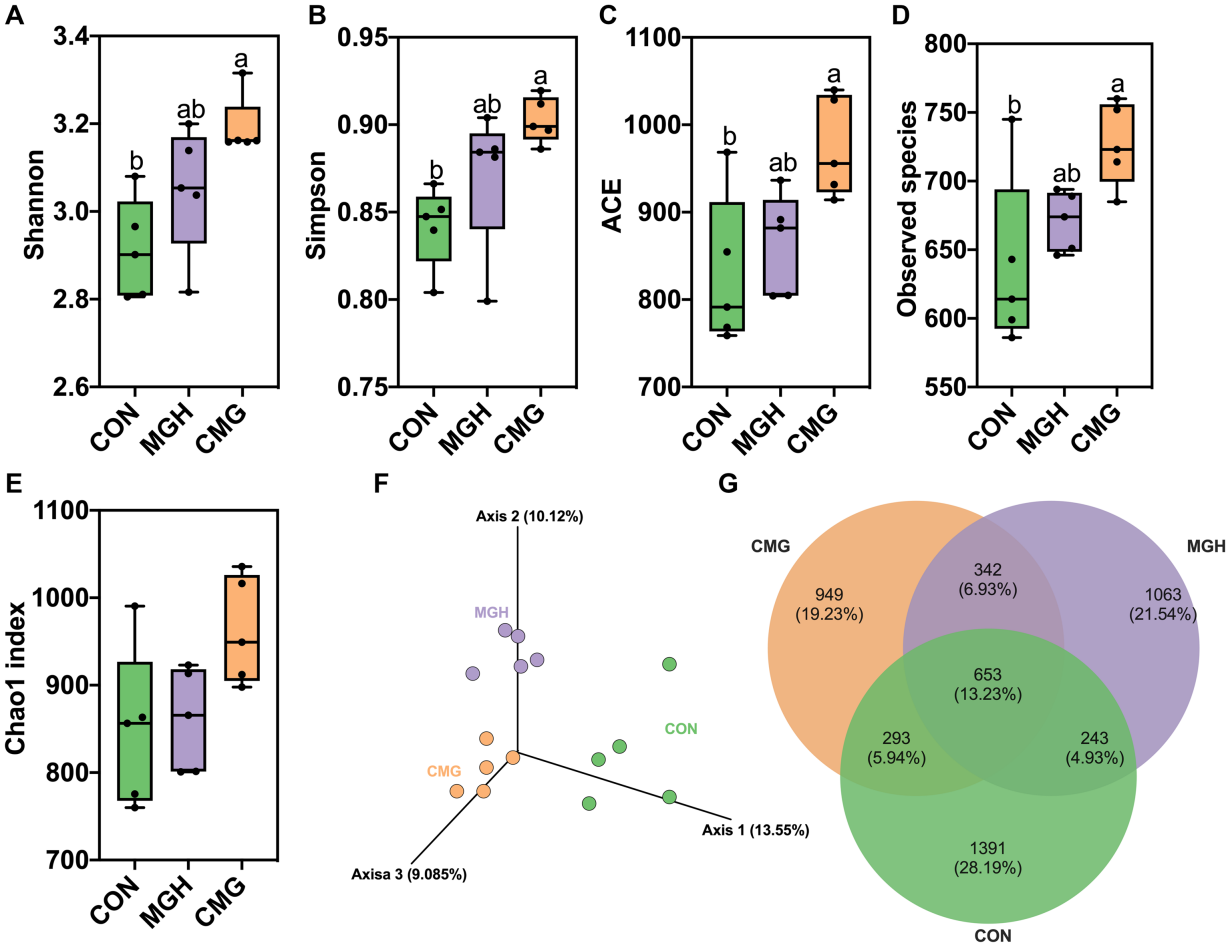
Effects of magnesium hydride (MGH) and coated-magnesium hydride (CMG) on the microbial community in the culture of *in vitro* fermentation using cecal inocula from broilers. **(A)** Shannon index; **(B)** Simpson index; **(C)** ACE; **(D)** Observed species; **(E)** Chao index; **(F)** Principal coordinate analysis (PCoA); and (G) Venn diagram of the common and unique OTUs among the CON, MGH and CMG groups. Different lowercase letters indicate significant differences (*p* < 0.05). CON, the control group, cecal content and feed; MGH, the magnesium hydride group, cecal content, feed and 200 mg/L MgH_2_; CMG, the coated- magnesium hydride group, cecal content, feed and 400 mg/L coated MgH_2_, (*n* = 5).

Rarefaction curves are useful for assessing the adequacy of sequencing data and indirectly reflect the richness of species in the samples. In Supplementary Figure S1A, the rarefaction curves for the observed operational taxonomic units (OTUs) approached a plateau, indicating that the sequencing depth was sufficient to cover the majority of OTUs present in the culture samples. Various measurement indicators, including Chao1, ACE, Shannon, and Simpson indices, were utilized to evaluate the effects of MGH and CMG on the microbial community structure. Surprisingly, compared to the CON group, the CMG group exhibited significantly increased values for observed species, Shannon, Simpson, and ACE indices, indicating higher species richness and diversity (*p* < 0.05) (Figures 3A–D). Conversely, MGH treatment had no significant impact on the observed species or the α diversity indices (*p* > 0.05) (Figures 3A–D). Additionally, there were no significant differences in the Chao1 index among the three experimental groups (Figure 3E).

#### β diversity of microbiota in culture

To evaluate the β diversity among the CON, MGH and CMG groups, the principal coordinate (PCoA) analysis (Figure 3F) and UPGMA clustering tree based on UniFrac (Supplementary Figure S1B) were plot at the OTU level. The unweighted Unifrac ANOSIM method (*R* = 0.6338, *p* = 0.001) demonstrated a significant distinction in microbial communities among the three experimental groups. Figure 3G shows that there were 1,391, 1,063, and 949 unique OTUs in the CON, MGH, and CMG groups, respectively, with 653 mutual OTUs among the three groups. Furthermore, Supplementary Figure S1B indicates that the three experimental groups cluster separately, suggesting that the addition of either MGH or CMG had a notable effect on the overall microbial community structure in the culture of *in vitro* fermentation by broiler cecal digesta.

#### Microbial species difference analysis

**Figure 4:**
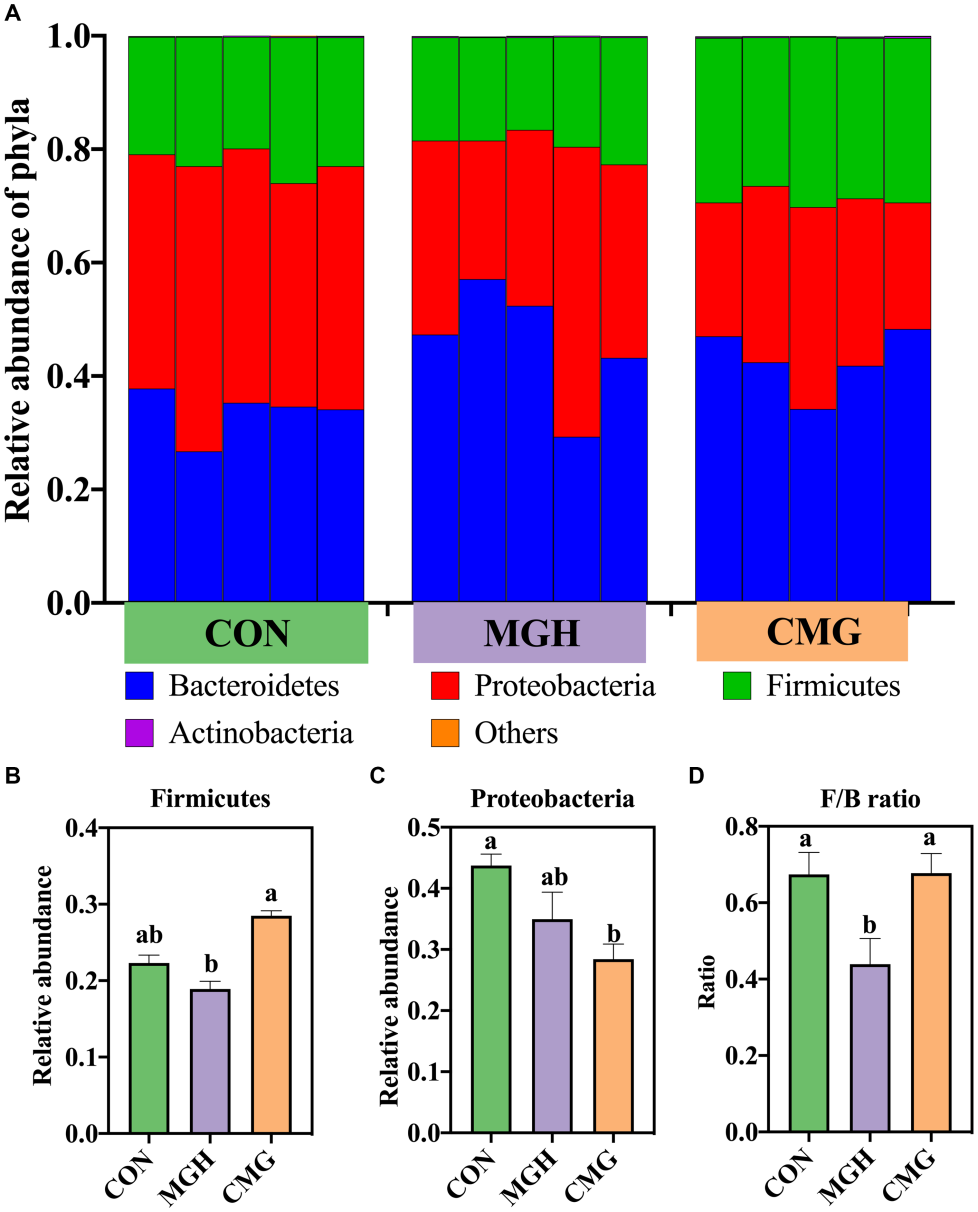
Effects of magnesium hydride (MGH) and coated-magnesium hydride (CMG) on bacterial community structure (phylum level) in the culture of *in vitro* fermentation using cecal inocula from broilers. **(A)** The top 4 dominant phyla relative abundances of bacterial strains (at the phylum level) among CON, MGH, and CMG groups; **(B,C)** The significant differences at phyla level which were compared by the Kruskal–Walli’s test among CON, MGH and CMG groups; **(D)** The ratio of the abundance of Firmicutes to Bacteroidetes at phyla level which were compared by one-way analysis of variance (ANOVA) with Duncan’s test among CON, MGH and CMG groups. Data are means ±SEM (5 replications). Different lowercase letters indicate significant differences (*p*<0.05). CON, the control group, cecal content and feed; MGH, the magnesium hydride group, cecal content, feed and 200 mg/L MgH_2_; CMG, the coated- magnesium hydride group, cecal content, feed and 400 mg/L coated MgH_2_.

To analyze the specific changes in microbial communities, the relative abundance of dominant taxa in the three experimental groups was examined. A total of 4 phyla and 168 genera were identified from 15 culture samples. Figure 4A illustrates the relative abundances of microbial taxa at the phylum level in the culture after 48 h of fermentation. The microbiota was primarily composed of Bacteroidetes (40.77%), Proteobacteria (35.71%), Firmicutes (23.24%), and Actinobacteria (0.18%). The differences in the composition of the microbiota at the phylum level among the three experimental groups was investigated (Figures 4B–D). The CMG group exhibited a higher relative abundance of Firmicutes and a higher Firmicutes/Bacteroidetes (F/B) ratio compared to the MGH group (*p* < 0.05). However, there was no significant difference between the CMG and MGH groups in terms of Firmicutes abundance or the F/B ratio (*p* > 0.05) (Figures 4B,D). On the other hand, the relative abundance of Proteobacteria in the CMG group was lower compared to the CON group (*p* < 0.05). However, no significant difference was observed between the CMG and MGH groups in terms of Proteobacteria abundance (*p* > 0.05) (Figure 4C).

**Figure 5:**
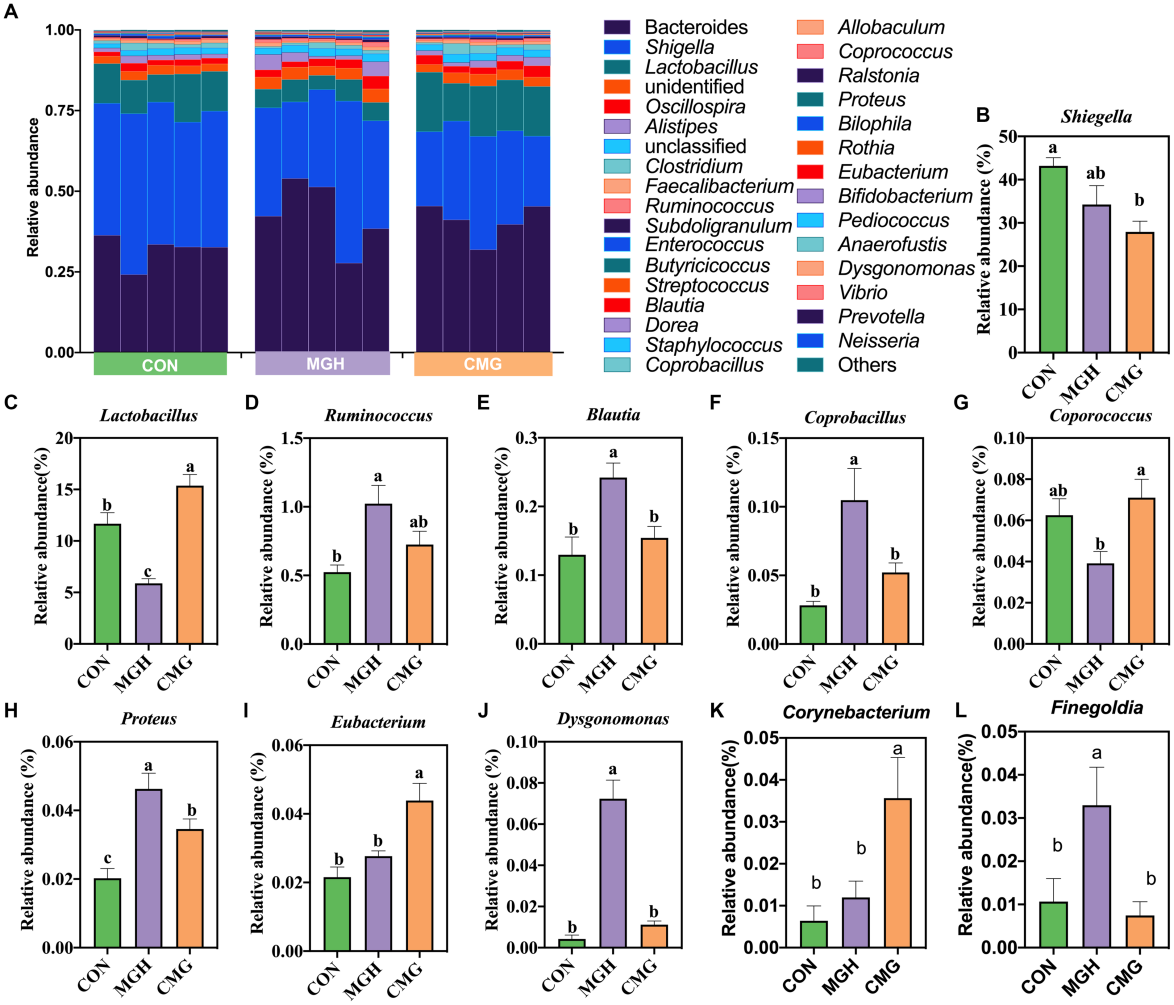
Effects of magnesium hydride (MGH) and coated-magnesium hydride (CMG) on microbial composition (genera level) in the culture of *in vitro* fermentation using cecal inocula from broilers. **(A)** The top 30 dominant genus relative abundances of bacterial strains (at the genera level) among CON, MGH and CMG groups; **(B–L)** The differences in the bacteria genera (relative abundance>0.01%) among CON, MGH and CMG groups which were compared by the Kruskal–Walli’s test. Data are means ±SEM (5 replications). Different lowercase letters indicate significant differences (*p* < 0.05). CON, the control group, cecal content and feed; MGH, the magnesium hydride group, cecal content, feed and 200 mg/L MgH_2_; CMG, the coated- magnesium hydride group, cecal content, feed and 400 mg/L coated MgH_2_.

Figure 5A presents the variations in the composition of the microbiota at the top 30 genus level. The 10 major classified genera across all treatments were *Bacteroides* (38.46%), *Shigella* (35.11%), *Lactobacillus* (10.95%)*, Oscillospira* (2.37%)*, Alistipes* (2.07%)*, Clostridium* (1.46%)*, Faecallibacterium* (0.87%)*, Ruminococcus* (0.76%)*, Subdoligranulum* (0.57%), and *Enterococcus* (0.51%) with a relative abundance greater than 0.5%. To better clarity the differences among the experimental groups, the differential microbiota analysis focused on genera with a relative abundance above 0.01%. Firstly, the relative abundance of *Shigella* in the MGH group did not differ significantly from that in the CON or CMG groups (*p >* 0.05) (Figure 5B). However, the CMG group have a lower abundance of *Shigella* compared to the CON group (*p <* 0.05) (Figure 5B). Notably, the relative abundance of *Lactobacillus* significantly differed among the three experimental groups (*p <* 0.05), with the order as follows: CMG *>* CON*>*MGH (Figure 5C). Additionally, compared to the CON group, MGH treatment increased the abundance of *Ruminococcus* (*p <* 0.05), while CMG addition had no significant impact on the level of *Ruminococcus* (*p >* 0.05) (Figure 5D). Furthermore, the relative abundance of *Blautia, Coprobacillus, Dysgonomonas*, and *Finegoldia* were significantly increased (*p* < 0.05) in MGH group compared to the CON and CMG groups, but no significant difference was found between the CON and CMG groups (*p* > 0.05) (Figures 5E,F,J,L). The relative abundance of *Coporococcus* in the MGH group was significantly lower than that in the CMG group (*p* < 0.05), while there was no significant difference compared to the CON group (*p >* 0.05) (Figure 5G). The level of Proteus also showed significant differences among the three experimental groups (*p* < 0.05), with the order as follows: MGH *>* CMG *>* CON (Figure 5H). Moreover, CMG treatment resulted a higher relative abundance of *Eubacterium* and *Corynebacterium* compared to the CON and MGH groups (*p* < 0.05), while no significant difference was observed between the CON and MGH groups (*p >* 0.05) (Figures 5I,K).

### Metabolomics analysis

#### Cluster analysis of metabolites in culture

To identify differentially expressed metabolites in the culture, the metabolomic profiling among the three experimental groups was compared. The scores plot derived from PLS-DA tests in both positive and negative metabolites displayed clearly distinct group separation (Supplementary Figures S2A,B). The scores plot from OPLS-DA tests also showed clear group separation (Supplementary Figures S2C,D), supporting the PLS-DA findings. Cross-validation and permutation tests validated the observed separation results with high reliability [OPLA-DA (−), Q^2^Y = 0.621; OPLS-DA (+), Q^2^Y = 0.605] (Supplementary Figures S2E,F).

#### Screening and identification of differential metabolites

**Figure 6:**
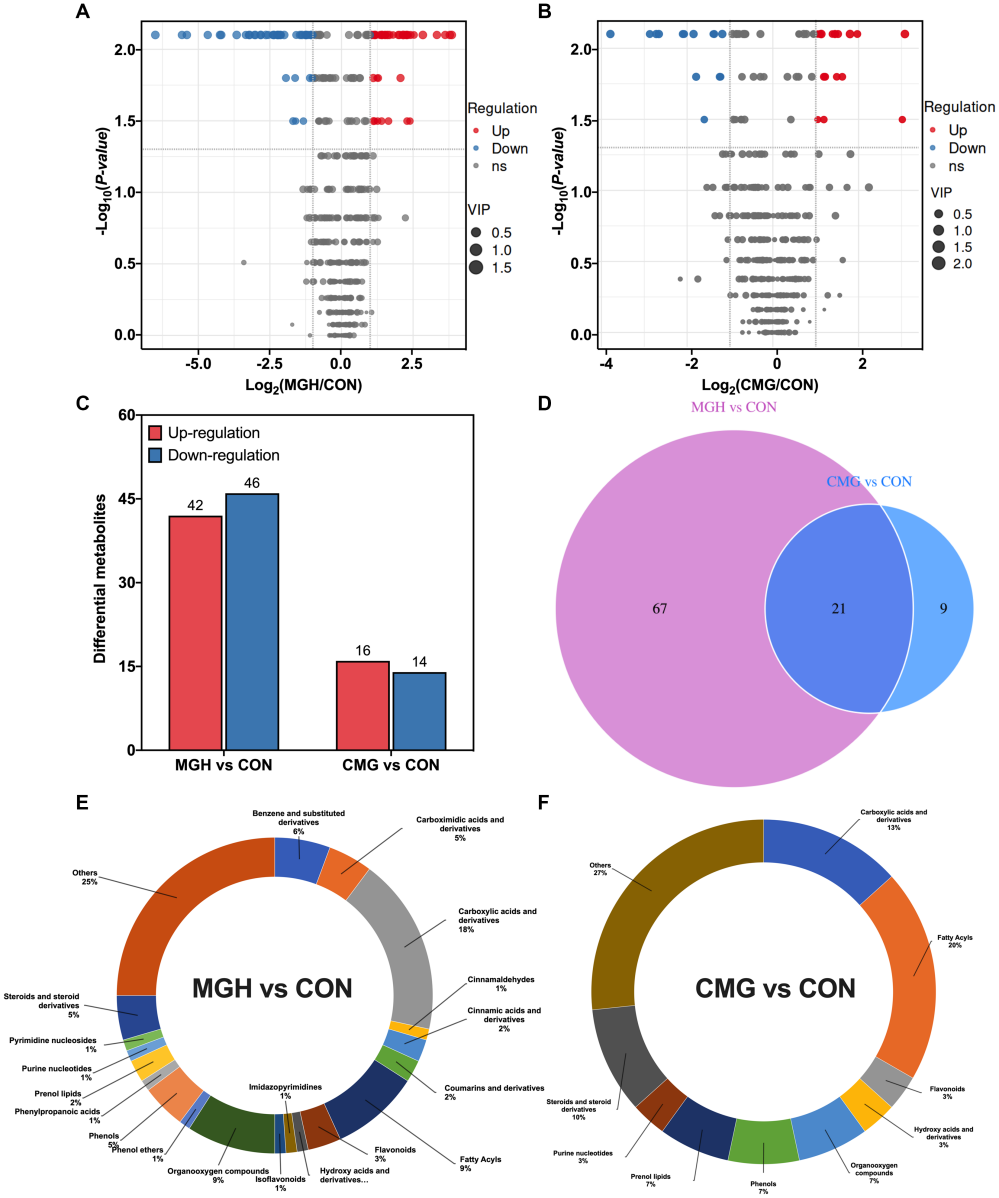
Effects of magnesium hydride (MGH) and coated-magnesium hydride (CMG) on the metabolome in the culture of *in vitro* fermentation using cecal inocula from broilers. **(A)** Score volcano plots of culture metabolomic data from MGH vs. CON groups; **(B)** Score volcano plots of culture metabolomic data from CMG versus CON groups; **(C)** The number of differentially expressed metabolites with functional annotations among CON, MGH, and CMG groups; **(D)** A Venn diagram was drawn to reveal the number of common and unique metabolites existing in the MGH vs. CON groups and CMG vs. CON groups; **(E)** Relative abundance of culture metabolites between MGH vs. CON groups; and **(F)** CMG vs. CON groups. CON, the control group, cecal content and feed; MGH, the magnesium hydride group, cecal content, feed and 200 mg/L MgH_2_; CMG, the coated- magnesium hydride group, cecal content, feed and 400 mg/L coated MgH_2_, (*n* = 5).

To screen for differentially metabolites among the groups, differential metabolites from two pairwise groups (MGH vs. CON and CMG vs. CON) with FC (fold change) > 2 or FC < 0.5, and *p* < 0.05 were visualized in the volcano plot (Figures 6A,B). The results presented a total of 88 (42 up-regulated, 46 down-regulated) and 30 (16 up-regulated and 14 down-regulated) differentially metabolites from the MGH vs. CON and CMG vs. CON pairwise groups (Figure 6C). Among the 88 differential metabolites from the comparison between the MGH and CON group, mainly include 16 carboxylic acid and derivatives, 8 fatty acyls, 8 organooxygen compounds, 5 benzene and substituted derivatives, 4 carboximidic acids and derivatives, 4 phenols and 4 steroids and steroid derivatives, and 22 unclassified metabolites (Figure 6E). The 30 differential metabolites from the comparison between CMG and CON groups, mainly include 6 fatty acyls, 4 carboxylic acids and derivatives, 3 steroids and steroids derivatives, and 8 unclassified metabolites (Figure 6F). The differential metabolites of the pairwise groups were selected and shown in heat maps (Supplementary Figures S2A,B), results suggested that the concentrations of core differential metabolites in the culture were significantly influenced either by MGH or CMG supplementations.

For the total 118 differential metabolites among the two pairwise groups (MGH vs. CON and CMG vs. CON), of which 21 were co-regulated metabolites, 67 were only regulated in the pairwise groups of MGH vs. CON, and 9 were only regulated in the pairwise groups of CMG vs. CON (Figure 6D). As summarized in Supplementary Table S1, the identified 21 biomarkers belong to the categories of Amino acids (4), Benzenoids (2), Carboxylic acids and derivatives (1), Lipids and lipid-like molecules (7), Nucleosides, nucleotides, and analogs (1), Organooxygen compounds (3), Phenylpropanoids and polyketides (1) and Others (2). Both MGH and CMG groups significantly increased the concentrations of phenylacetylglutamine, linatine, fumaric acid, alpha-linolenic acid, corticosterone, 2-methoxyestrone acetylcholine chloride, *D*-Ribose and apigenin (mainly belonging to amino acids and lipids and lipid-like molecules) in the culture when compared with the CON group (*p* < 0.05). Moreover, MGH and CMG groups also significantly decreased the culture concentrations of 2-amino-2-deoxy-*D*-gluconate, vanillylmandelic acid, 3,4-Dihydroxymandelic acid, estradiol, isoalantolactone, costunolide, citramalic acid, GMP, 2-Oxo-4-phenylbutyric acid, myo-Inositol, and 3-ketosphingosine when compared with the CON group (*p <* 0.05).

#### Analysis of metabolic KEGG pathway

**Figure 7:**
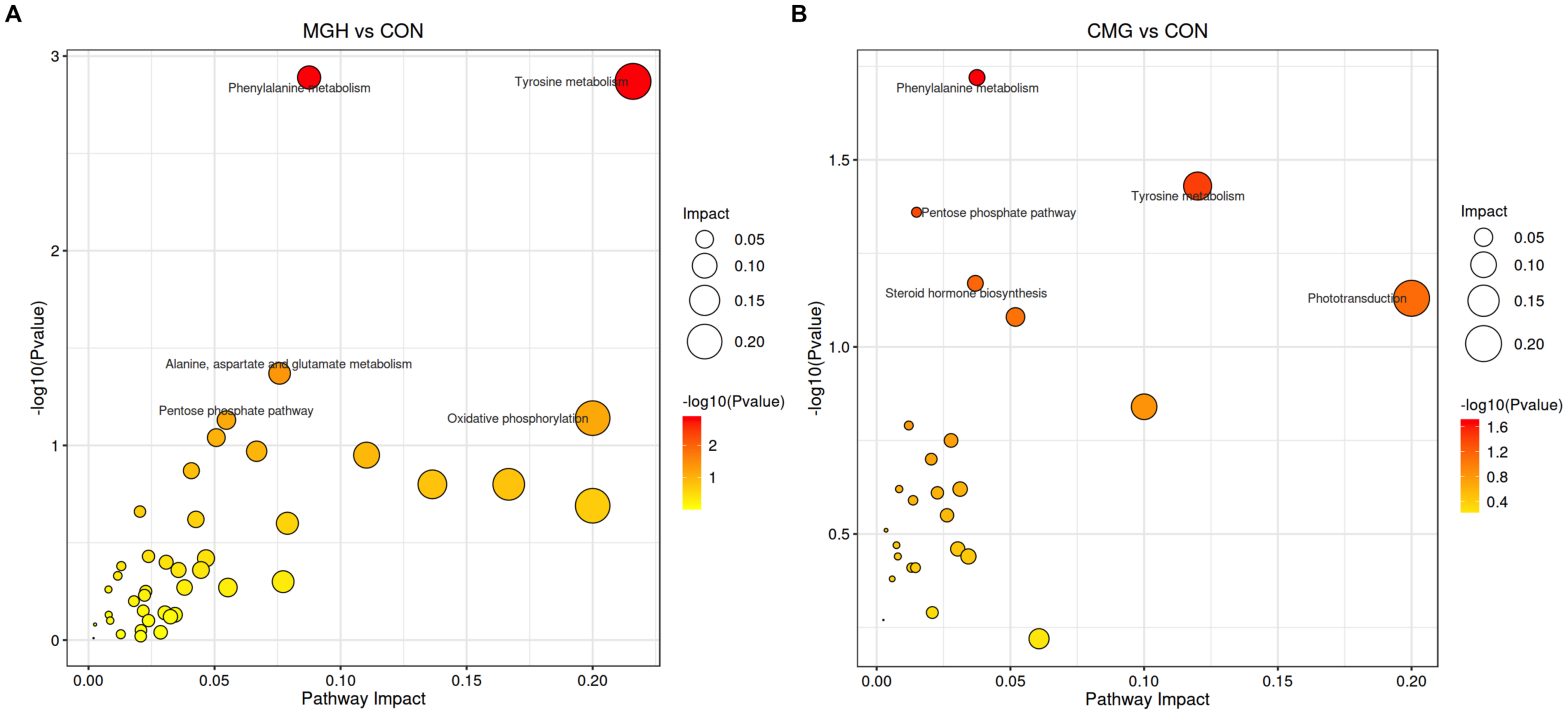
Effects of magnesium hydride (MGH) and coated-magnesium hydride (CMG) on the metabolic pathways in the culture of *in vitro* fermentation using cecal inocula from broilers. **(A)** MGH vs. CON groups; **(B)** CMG vs. CON groups. CON, the control group, cecal content and feed; MGH, the magnesium hydride group, cecal content, feed and 200 mg/L MgH_2_; CMG, the coated- magnesium hydride group, cecal content, feed and 400 mg/L coated MgH_2_, (*n* = 5).

Furthermore, KEGG analysis was performed to analyze the pathways associated with differentially abundant metabolites among the three experimental groups. For the MGH vs. CON pairwise comparison, three metabolic pathways, namely phenylalanine metabolism, tyrosine metabolism, and alanine, aspartate, and glutamate metabolism, were found to be significantly affected by MGH (Figure 7A, *p* < 0.05). Similarly, for the CMG vs. CON pairwise comparison, the three metabolic pathways of the phenylalanine metabolism, tyrosine metabolism, and pentose phosphate pathway were found significantly affected by CMG (Figure 7B, *p* < 0.05).

#### Correlation analysis involving hydrogen production, microbiota and metabolites

**Figure 8:**
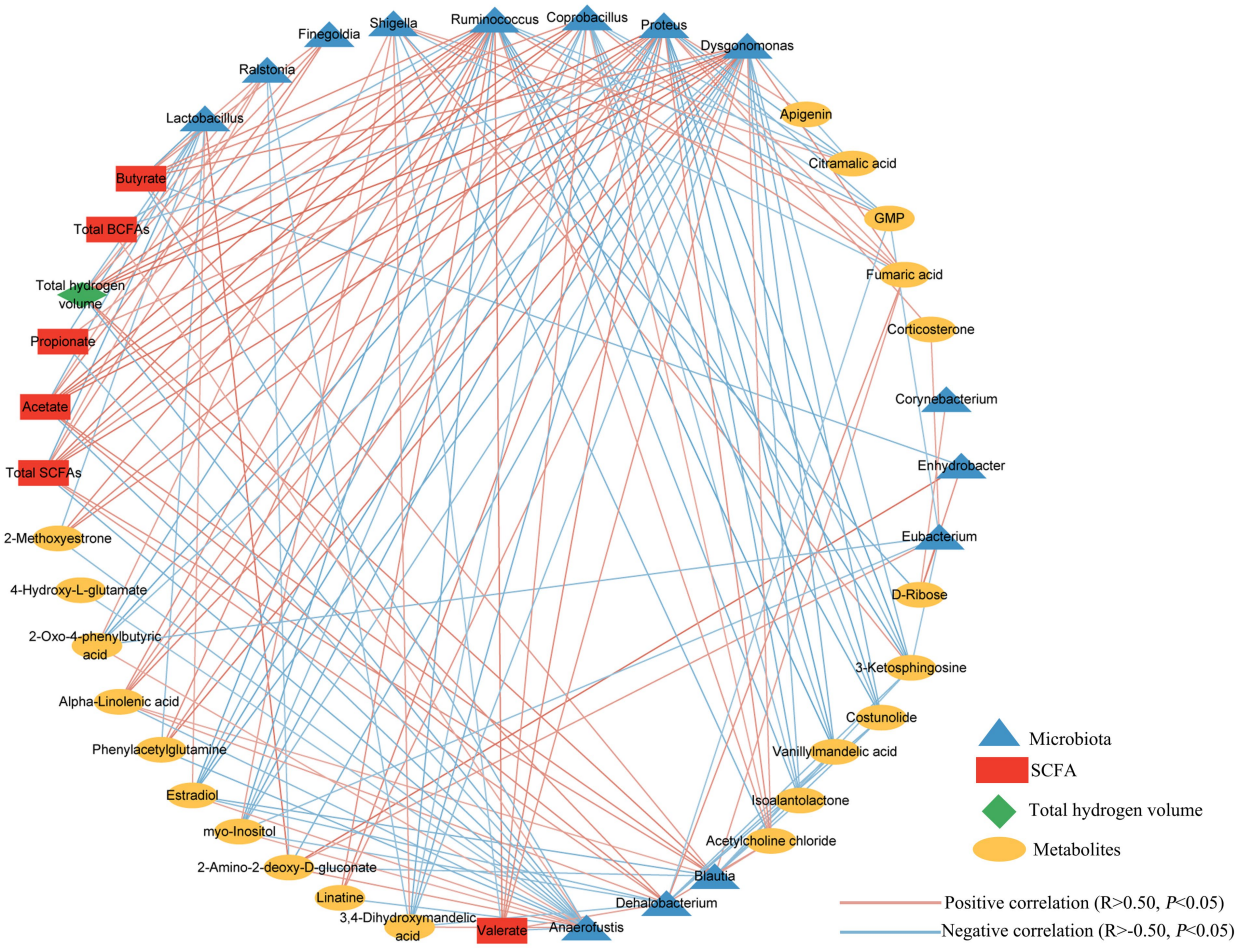
Correlation network involving total hydrogen gas volume, differentially changed microbiota (at genus level), and metabolites. Each line has an absolute Spearman rank correlation; orange lines represent positive correlation (*R* > 0.50, *p* < 0.05); and blue lines represent negative correlation (*R* < –0.50, *p* < 0.05).

To further investigate relationship involving the microbiota, metabolites and total hydrogen production in the culture, we performed Spearman’s rank correlation coefficients analysis (Figure 8). Our analysis revealed significant correlations between the total hydrogen volume and several genera, including *Finegoldia*, *Ralstonia*, *Ruminococcus*, *Coprobacillus*, *Proteus*, *Dysgonomonas*, *Blautia*, and *Dehalobacterium,* all of which showed a positive correlation. Conversely, the genera of *Anaerofustis* and *Lactobacillus* exhibited a negative correlation with the total hydrogen gas volume. Interestingly, we also observed a significant negative correlation between hydrogen production and levels of propionate and total BCFAs. Furthermore, we observed strong correlations between core metabolites and specific differential genera of microbiota. Taken together, these results suggest that the supplementation of exogenous hydrogen by MGH or CMG altered the microbiota composition, leading to changes in metabolites during *in vitro* fermentation using broiler cecal as inoculum in the culture.

## Discussion

Gas production through *in vitro* fermentation is commonly used as an indicator to measure the breakdown of dietary nutrition by intestinal flora in animals (Newbold et al., 2005). In our study, we observed that the MGH group exhibited rapid and intense fermentation patterns, as evidenced by the highest cumulative gas productions, *T*_1/2_ and *R*_max_, along the lowest *T*_Rmax_ compared to the CON group. This suggests that MGH influenced the fermentation process and may serve as a supplementary nutrient source for growth and proliferation of microbiota. Notably, the MGH group exhibited the highest total hydrogen gas production among the three groups, followed by the CMG group, while the CON group showed minimal hydrogen production. This indicates that the coated-MGH not only functions as a delayed-release compound but may also promote cecal microflora fermentation (Bernard et al., 1999). Interestingly, in our study, the measured hydrogen gas production in the MGH group exceeded the theoretical maximum yield of magnesium hydride added. MGH rapidly generates hydrogen gas upon contact with water (Lee et al., 2020), and the microbial community during fermentation also produces hydrogen by metabolizing carbohydrates (Smith et al., 2019). This suggests that high concentrations of external hydrogen gas may stimulate microbial consumption of carbohydrates, leading to increase hydrogen gas production. Consistent with our findings, previous research has proposed that the consumption of hydrogen by methanogens enhances the efficiency of carbohydrate metabolism by the microbiota, resulting in the increased concentrations of SCFAs (Turnbaugh et al., 2006). The lower hydrogen gas production in the CMG group may be attributed to the fact that the MGH component, which forms the outer layer of coated MGH, hinders direct contact between MGH and water (Chao, 2018).

After 48 h of incubation, the MGH group exhibited a decrease in N-NH_3_ content compared to the CON and CMG groups. High levels of SCFAs and low levels of N-NH_3_ are generally considered beneficial for long-term host health, while low levels of SCFAs and high levels of N-NH_3_ are considered detrimental (Guo et al., 2003). Additionally, the CMG group showed an increase in lactic acid content compared to the CON and MGH groups. Lactic acid can lower the pH in the gut, creating an unfavorable environment for pathogenic bacteria (Dittoe et al., 2018). This differences in pH may explain the variation between the MGH and CMG groups in this study. The main byproducts of carbohydrate fermentation were SCFAs, which are typically associated with beneficial effects on host health (Liu et al., 2021). In our study, the MGH group exhibited a significant increase in acetate, propionate, butyrate, valerate, and total SCFAs, along with a decrease BCFAs compared to the CON group. This aligns with our findings in piglets fed a mycotoxin-contaminated diet, where the consumption of HRW (0.60 mM) for 25 days resulted in elevated levels of butyric acid, valeric acid, and total SCFAs in the large intestine (Zheng et al., 2018). On the other hand, the CMG group also showed a significant increase in acetic acid, valerate acid, as well as a decrease in BCFAs compared to the CON group. However, the CMG group exhibited significantly lower levels of acetic acid, valerate acid, and total SCFAs compared to the MGH group. BCFAs are derived from branched-chain amino acids (such as valine, leucine, and isoleucine). Excessive fermentation of amino acid or protein in the hindgut can result in the production of nitrogenous metabolites that can harm intestinal integrity (Russell et al., 2011). The differences in SCFAs production may be attributed to variations in hydrogen concentrations within the culture. Studies have indicated that high hydrogen concentrations can enhance the metabolic efficiency of specific SCFA-producing bacteria, leading to increased production of SCFAs (Smith et al., 2019; Ge et al., 2022). Indeed, our correlation analysis revealed a negative correlation between hydrogen production with levels of propionate and total BCFAs (Figure 8). This finding provides further evidence to support the relationship between hydrogen production and SCFAs. Taken together, our findings demonstrated that MGH and CMG supplementation can improve broiler cecal metabolism in distinct manners during *in vitro* fermentation.

The intestinal microbiota plays a crucial role in various aspects of host health, including physiology, immunity, nutrient absorption, and metabolism (Bindari and Gerber, 2022). Based on the combined results of our α diversity and β diversity, we found that CMG supplementation had a significant impact on the composition of the microbiome in the fermentation culture using broiler cecal digesta. Unfortunately, our results did not indicate a significant difference in α diversity within the MGH group, suggesting MGH may act on specific types of bacteria rather than a wide range. Specifically, we observed that the CMG group had the highest relative abundance of Firmicutes among the three groups, while the relative abundance of Proteobacteria was significantly lower in the CMG group compared to the CON group. This aligns with previous findings, where hydrogen-rich water treatment for 20 days improved the relative abundance of Firmicutes and decreased the relative abundance of Proteobacteria in mice feces (Lian et al., 2021). Firmicutes and Bacteroidetes are the predominant bacterial populations in the chicken gut, known for their role in nutrients digestion, absorption, and maintaining a stable digestive tract environment (Clavijo and Florez, 2018). In addition, studies have shown that members of the Firmicutes and Bacteroidetes are primary producers of hydrogen gas in the human colon (Carbonero et al., 2012). High hydrogen concentrations can disrupt the metabolism of both hydrogen-producing and non-producing bacteria (Smith et al., 2019). Therefore, the relatively lower F/B ratio observed in the MGH group may be attributed to high hydrogen concentration produced by MGH, which alters the balance of the microbial hydrogen metabolism. Proteobacteria, comprising gram-negative bacteria, includes various pathogenic species such as *Escherichia* spp.*, Campylobacter* spp., and *Salmonella* spp. (Orso et al., 2021). Remarkably, the CMG group demonstrated a reduction in the relative abundance of *Shigella*, a pathogenic bacterium belonging to the Proteobacteria phylum. Studies have shown that under stress conditions, *Shigella* can become pathogenic, leading to increased intestinal permeability, disrupted epithelial barriers, and intestinal diseases (Ribet and Cossart, 2015). Therefore, CMG supplementation may attribute to improve poultry health and food safety by reducing the abundance of these pathogenic bacteria in the intestine.

At the genus level, the MGH group exhibited a higher relative abundance of *Blautia*, *Ruminococcus*, *Dysgonomonas*, and *Coprobacillus* compared to the CON and CMG groups. Additionally, the CMG groups showed a significant higher relative abundance of *Lactobacillus*, *Eubacterium*, and *Coprococcus* compared to the CON and MGH groups. *Lactobacillus* is widely regarded as a beneficial bacterium that helps maintain a healthy balance of gut microbiota and prevents the invasion of pathogens (Angelakis and Raoult, 2010). *Eubacterium* has a significant role in reducing systemic inflammation by preserving the integrity of the gut barrier and preventing the invasion of pathogenic microbes and toxins (Singh et al., 2022). *Coprococcus*, on the other hand, is an important produce of butyrate, which plays a role in evaluating the gastrointestinal tract’s health as a microbial biomarker (Zhang et al., 2019). *Dysgonomonas* is capable of degrading various polysaccharides and complex compounds, including lignocellulose, and has been implicated in the generation of SCFAs production (Martínez-Oca et al., 2020; Bridges and Gage, 2021). The higher abundance of *Dysgonomonas* and *Coprobacillus* in the MGH groups may responsible for the increased production of SCFAs observed in the culture (Figure 2). In contrast, the CMG demonstrated the ability to change the relative abundance of beneficial microorganisms while reducing the presence of pathogenic bacteria.

Our correlation analysis involving hydrogen gas production, microbiota and metabolites revealed positive associations between the total hydrogen gas production and the abundance of several genera including *Ruminococcus*, *Blautia*, *Coprobacillus*, *Dysgonomonas*, *Finegoldia*, *Proteus*, *Dehalobacterium*, and *Ralstonia* (Figure 8). Conversely, negatively correlation was observed with *Anaerofustis*, *Lactobacillus*, total BCFAs, and propionate (Figure 8). This aligns with previous studies demonstrating the influence of various bacteria on hydrogen gas production. For instance, *Ruminococcus* has been shown to produce high concentrations of H_2_ *in vitro* (Simmering et al., 2002). *Blautia* is involved in the non-functional Wood-Ljungdahl pathway (WLP) of H_2_ + CO_2_ acetylation, where hydrogen serves as an electron donor to reduce carbon dioxide to acetic acid (Trischler et al., 2022). *Eubacterium* is also a well-known bacterium that produce H_2_ (Kalantar-Zadeh et al., 2019)_._ Additionally, previous research has suggested that molecular hydrogen can promote the growth of SCFA-producing bacteria such as *Ruminococcus* and *Blautia*, thereby supporting intestinal health (Higashimura et al., 2018). Despite the observed differences in microbial composition between the MGH and CMG groups, our results also demonstrate significant impact of exogenous hydrogen on the microbiota in the fermentation culture using broiler caecal as inoculum.

Metabolome profiles play a critical role in detecting illness, guiding treatments, and assessing the nutritional status of broilers. For instance, fumaric acid has been identified as a potential agent for the improvement intestinal histomorphology and has shown effectiveness when incorporated into broiler diets (Yang et al., 2018; Abdelli et al., 2021). In our study, the addition of MGH and CMG had a co-regulatory effect on 21 metabolites in the culture (Supplementary Table S1). The MGH and CMG groups exhibited significant higher levels of fumaric acid, with respective fold changes of 4.44 and 3.49 compared to the CON group. Analysis of the KEGG metabolic pathways revealed that the differentially expressed metabolites in the culture were primarily associated with carbohydrate and amino acid metabolism. Specifically, both MGH and CMG treatments significantly enhanced phenylalanine and tyrosine metabolism, which are known to be closely linked to oxidative stress response (Ipson et al., 2019). Our findings are consistent with a previous study that reported significant alternations in the fecal microbiota of rats following hydrogen gas treatment, leading to increased phenylalanine, tyrosine and tryptophan biosynthesis (Xie et al., 2022). The gut microbiome plays a role in regulating intestinal functions through its metabolites, including hydrogen gas and SCFAs. In this study, our objective was to explore the interrelationship between hydrogen gas production, co-regulated microbiota, and metabolites influenced by exogenous hydrogen supplementation using MGH or CMG. Specifically, we found that *Ruminococcus* and *Blautia* were negatively associated with reactive oxygen species-related metabolites such as isoalantolactone and costunolide (Wang et al., 2016; Lu et al., 2018). In contrast, *Coprobacillus* and *Ruminococcus* showed a positive association with fumaric acid and alpha-Linolenic acid. Alpha-Linolenic acid demonstrated antioxidant effects by reducing lipid peroxidation and restoring antioxidant enzymes such as superoxide dismutase, glutathione peroxidase, and catalase (Pal and Ghosh, 2012). Interestingly, we found that total gas production did not exhibit a significant correlation with co-regulated metabolites. However, it did show a significant correlation with certain bacterial genera in the culture. Therefore, the joint analysis of the total hydrogen production, 16S rRNA gene sequencing and untargeted metabolomic revealed that MGH or CMG treatments had a direct impact on the microbiota, which in turn indirectly influenced metabolites in the culture. Our research provides strong evidence for further investigating the effects of MGH on the cecal microecology of broilers. However, it is important to acknowledge the significant limitations of our study. Further studies should focus on examining the effects of CMG supplementation on growth performance, microbiota and intestinal health in broilers *in vivo*.

## Conclusion

In summary, our results demonstrated that both MGH and CMG groups exhibited similar effects on fermentation properties, with increased gas and hydrogen gas production in the *in vitro* broiler caeal incubations. Specifically, MGH supplementation resulted in decreased N-NH_3_ content, and higher production of SCFAs. On the other hand, CMG supplementation appeared to enhance bacterial fermentation, leading to increased production of lactic acid, acetic acid, and valeric acid. Furthermore, MGH supplementation was found to significantly improve the abundance of SCFA-producing bacteria, while CMG promoted the relative abundance of beneficial microorganisms and reduced the pathogenic bacteria. Additionally, both MGH and CMG were associated with co-regulation of 21 differentially expressed metabolites (such as fumaric acid, and Alpha-Linolenic acid) and prominent KEGG pathways (phenylalanine metabolism and tyrosine metabolism). Moreover, joint correlation analysis revealed that MGH or CMG treatments had a direct impact on the microbiota, which in turn indirectly influenced metabolites in the culture.

## Data availability statement

The datasets presented in this study can be found in the NCBI Sequence Read Archive accession PRJNA924981.

## Ethics statement

The animal study was approved by Nanjing Agricultural University Animal Care and Use Committee (Nanjing, China). The study was conducted in accordance with the local legislation and institutional requirements.

## Author contributions

HH implemented the study and wrote the manuscript. HH, HZ, and HY assisted in conducting the experiment and collecting samples. WY and WZ designed the experiment. WZ revised the manuscript. All authors contributed to the article and approved the submitted version.

## Funding

This work was supported by the National Key R&D Program of China (2017YFE114400) and supported by the Center of Hydrogen Science, Shanghai Jiao Tong University.

## Acknowledgments

The author would like to thank Hangzhou King Techina Technology Company for providing coating materials. The author also would like to express their gratitude Honglin Jiang from Virginia Tech for providing valuable guidance and advise during preparing and revision of this manuscript.

## Conflict of interest

The authors declare that the research was conducted in the absence of any commercial or financial relationships that could be construed as a potential conflict of interest.

## Publisher’s note

All claims expressed in this article are solely those of the authors and do not necessarily represent those of their affiliated organizations, or those of the publisher, the editors and the reviewers. Any product that may be evaluated in this article, or claim that may be made by its manufacturer, is not guaranteed or endorsed by the publisher.

## Footnotes

^1^ http://www.metaboanalyst.ca

^2^ http://www.kegg.jp

^3^ https://www.omicstudio.cn/tool
