# Supplementary material for: In vitro fermentation properties of magnesium hydride and related modulation effects on broiler cecal microbiome and metabolome
Source: Front Microbiol. 2023 Aug 9;14:1175858. doi: 10.3389/fmicb.2023.1175858 (PMC10445219; doi:10.3389/fmicb.2023.1175858)
Supplement: Supplementary file 1 [file Data_Sheet_1.docx]

Supplementary Material

*In vitro* fermentation properties of magnesium hydride and related modulation effects on broiler cecal microbiome and metabolome

Heng Hu^1^, Zhu He^1^, Haiyan Yang^2^, Wen Yao^1,3^, Weijiang Zheng^1*^

^1^College of Animal Science and Technology, Nanjing Agricultural University, Nanjing, Jiangsu, China 210095

^2^ Center of Hydrogen Science, Shanghai Jiao Tong University, Shanghai, 200240, China

^3^Key lab of Animal Physiology and Biochemistry, Ministry of Agriculture, Nanjing, Jiangsu, China 210095

*** Correspondence:** Weijiang Zheng, zhengweijiang@njau.edu.cn

**Supplementary Table S1** Comparisons of culture metabolic biomarkers among CON, MGH and CMG groups (n=5).

| Metabolites | Formula | Category | MGH^2^ vs CON^1^ | | CMG^3^ vs CON^1^ | |
| --- | --- | --- | --- | --- | --- | --- |
|  |  |  | FC^4^ | *P*-value^5^ | FC^4^ | *P*-value^5^ |
| 2-Amino-2-deoxy-D-gluconate | C_6_H_13_NO_6_ | Amino acids | 0.099 | 0.034 | 2.064 | 0.016 |
| Phenylacetylglutamine | C_13_H_16_N_2_O_4_ | Amino acids | 12.256 | 0.013 | 2.815 | 0.027 |
| 4-Hydroxy-L-glutamate | C_5_H_9_NO_5_ | Amino acids | 13.766 | 0.009 | 8.094 | 0.2 |
| Linatine | C_10_H_17_N_3_O_5_ | Amino acids | 5.325 | 0.001 | 3.071 | 0.012 |
| Vanillylmandelic acid | C_9_H_10_O_5_ | Benzenoids | 0.079 | ＜0.001 | 0.38 | 0.015 |
| 3,4-Dihydroxymandelic acid | C_8_H_8_O_5_ | Benzenoids | 0.011 | 0.005 | 0.137 | 0.007 |
| Fumaric acid | C_4_H_4_O_4_ | Carboxylic acids and derivatives | 4.440 | 0.009 | 3.49 | 0.002 |
| Estradiol | C_18_H_24_O_2_ | Lipids and lipid-like molecules | 0.039 | 0.001 | 0.422 | 0.014 |
| Isoalantolactone | C_15_H_20_O_2_ | Lipids and lipid-like molecules | 0.021 | 0.002 | 0.239 | 0.012 |
| Costunolide | C_15_H_20_O_2_ | Lipids and lipid-like molecules | 0.194 | ＜0.001 | 0.386 | 0.002 |
| Alpha-Linolenic acid | C_18_H_30_O_2_ | Lipids and lipid-like molecules | 3.487 | 0.029 | 2.071 | 0.027 |
| Corticosterone | C_21_H_30_O_4_ | Lipids and lipid-like molecules | 3.122 | 0.001 | 3.93 | 0.008 |
| 2-Methoxyestrone | C_19_H_24_O_3_ | Lipids and lipid-like molecules | 4.954 | 0.001 | 2.336 | 0.006 |
| Citramalic acid | C_5_H_8_O_5_ | Lipids and lipid-like molecules | 0.429 | ＜0.001 | 0.441 | ＜0.001 |
| GMP | C_10_H_14_N_5_O_8_P | Nucleosides, nucleotides, and analogues | 0.024 | 0.006 | 0.072 | 0.001 |
| 2-Oxo-4-phenylbutyric acid | C_10_H_10_O_3_ | Others | 0.17 | ＜0.001 | 0.234 | ＜0.001 |
| Acetylcholine chloride | C_7_H_16_NO_2_. Cl | Others | 2.273 | ＜0.001 | 2.181 | ＜0.001 |
| D-Ribose | C_5_H_10_O_5_ | Organooxygen compounds | 3.97 | ＜0.001 | 8.484 | ＜0.001 |
| Myo-Inositol | C_6_H_12_O_6_ | Organooxygen compounds | 0.225 | ＜0.001 | 0.278 | ＜0.001 |
| 3-Ketosphingosine | C_18_H_35_NO_2_ | Organooxygen compounds | 0.053 | ＜0.001 | 0.159 | ＜0.001 |
| Apigenin | C_15_H_10_O_5_ | Phenylpropanoids and polyketides | 2.660 | ＜0.001 | 2.734 | 0.003 |

^1^CON, the control group, cecal content and feed

^2^MGH, the magnesium hydride group, cecal content, feed and 200mg/L of MgH_2_

^3^CMG, the coated-magnesium hydride group, cecal content, feed and 400mg/L of coated MgH_2_

^4^FC, fold change

^5^*P*-value, *P*-value calculated by Student’s t test

**
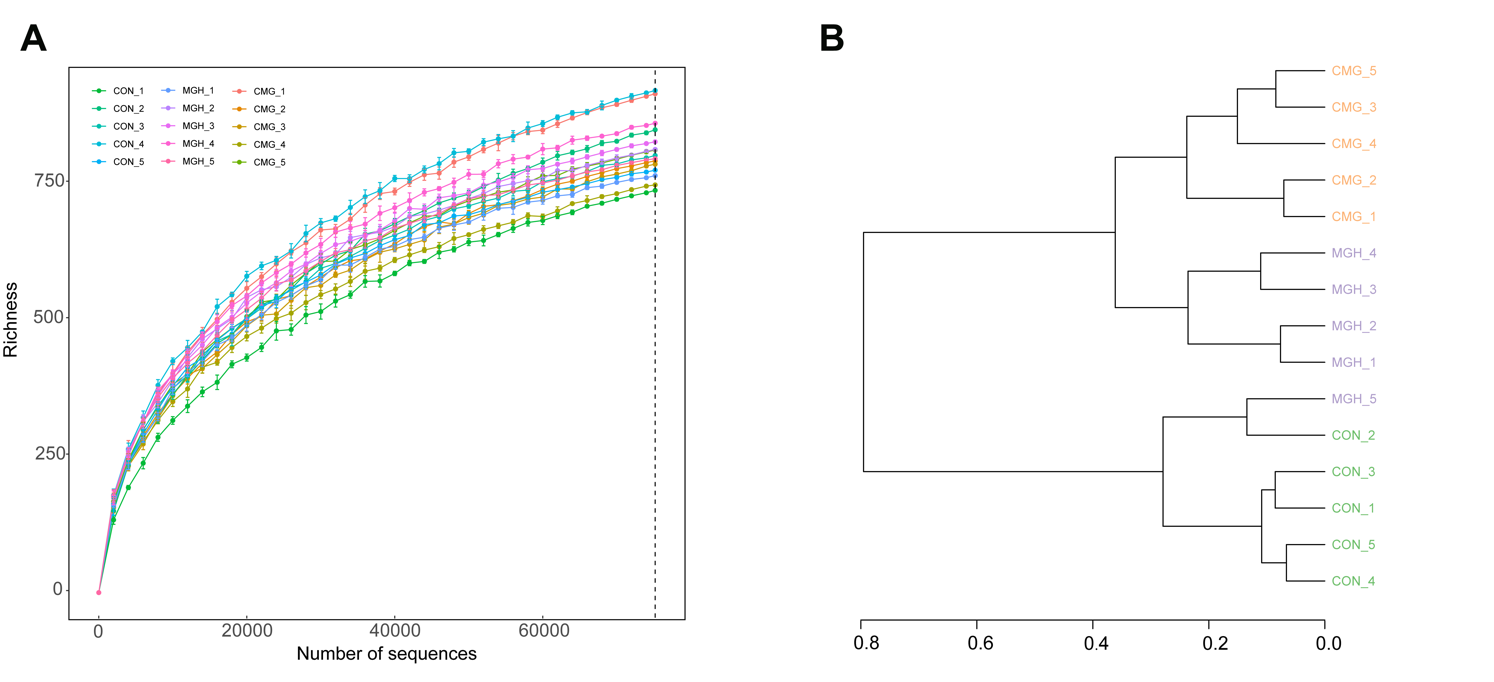
Supplementary Figure S1.** The alpha rarefaction (A) of all samples, and UPGMA clustering analysis of different treatments based on weighted UniFrac distance(B).


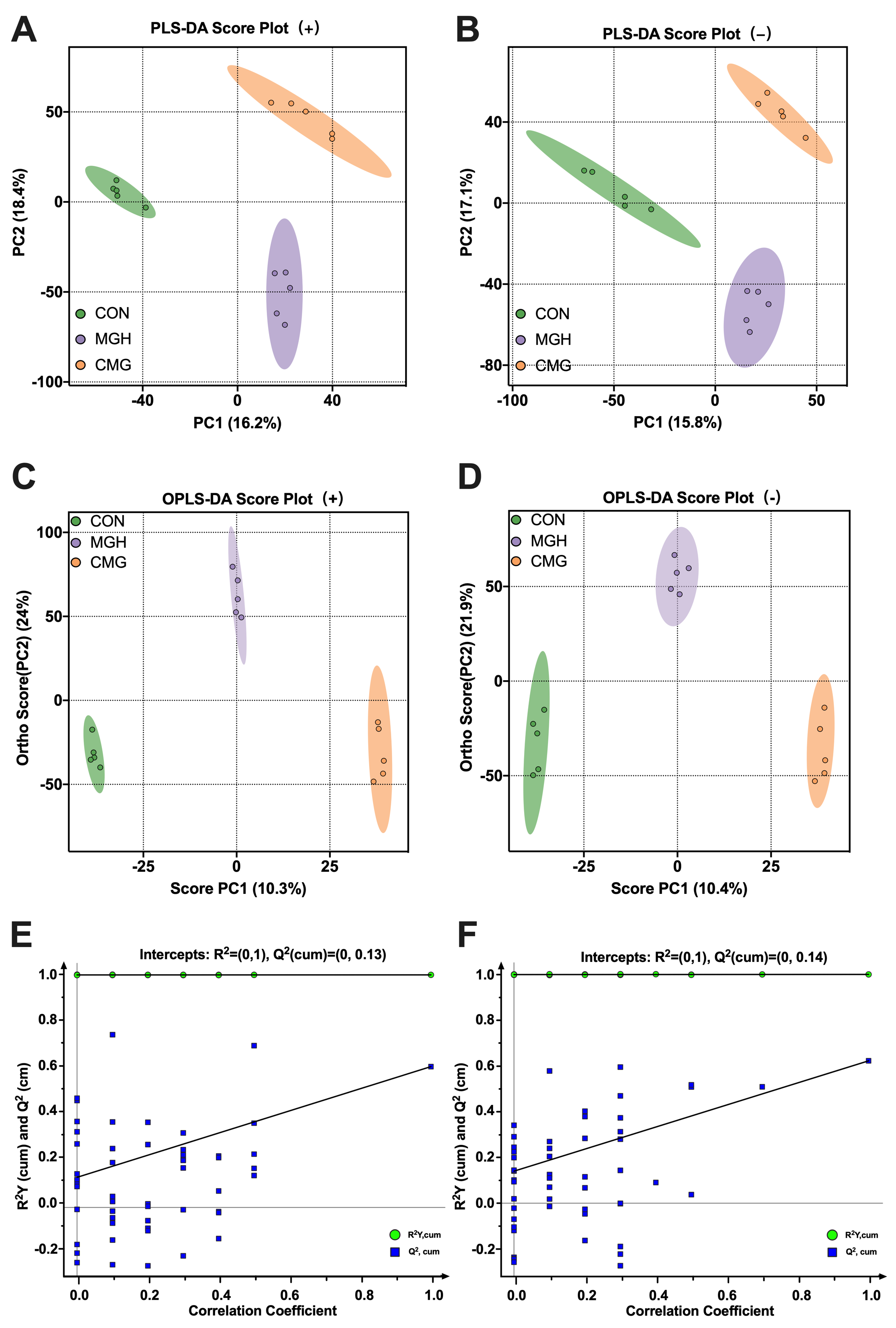


**Supplementary Figure S2.** Effects of magnesium hydride (MGH) and coated-magnesium hydride (CMG) on the microbial metabolites of culture by *in vitro* fermentation using broiler caecal as inoculum. (A) PLS-DA analysis of positive ions among CON, MGH and CMG groups; (B) PLS-DA analysis of negative ions among CON, MGH and CMG groups; (C) OPLS-DA analysis of positive ions among CON, MGH and CMG groups; (D) OPLS-DA analysis of negative ions among CON, MGH and CMG groups; and (E and F) The corresponding coefficient of loading plots of positive and negative ions among CON, MGH and CMG groups. CON, the control group, cecal content and feed; MGH, the magnesium hydride group, cecal content, feed and 200mg/L MgH_2_ CMG, the coated- magnesium hydride group, cecal content, feed and 400 mg/L coated MgH_2_, (n=5).


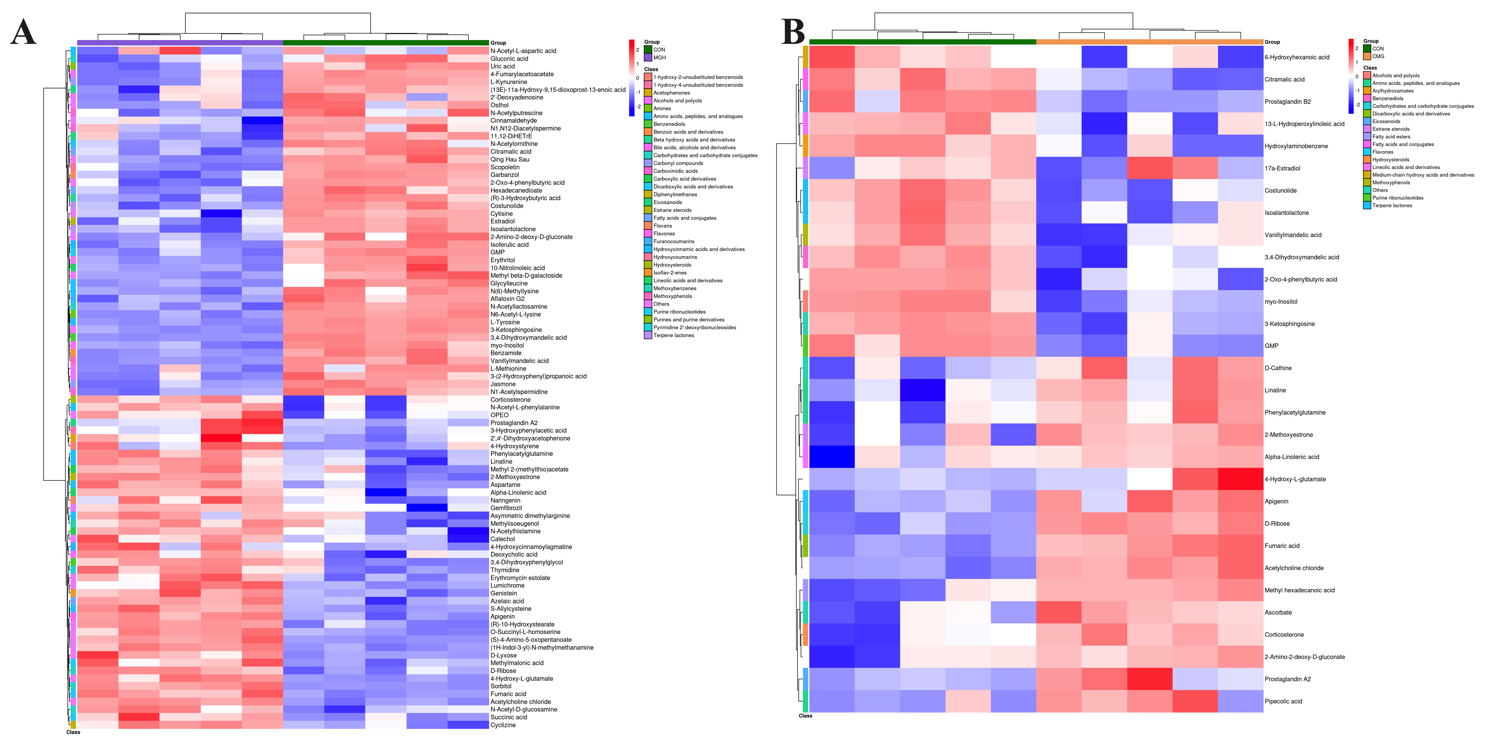


**Supplementary Figure S3.** Heat map visualizing differential metabolites and their similarity in MGH vs CON groups (A) and CMG vs CON groups (B) by hierarchical clustering analysis. CON, the control group, cecal content and feed; MGH, the magnesium hydride group, cecal content, feed and 200mg/L MgH_2_; CMG, the coated- magnesium hydride group, cecal content, feed and 400 mg/L coated MgH_2_, (n=5).
